# Poisoning by Sulphate of Iron

**Published:** 1844-08-24

**Authors:** 


POISONING BY SULPHATE OF IRON.

A case of considerable importance in a medico-legal point of view, is reported in the *London and Edinburg Medical Journal*, as having occurred to Dr. Christison. A child, four years of age, having died in circumstances which excited strong suspicions of poisoning, an investigation was made by the law authorities nearly four months afterwards. The symptoms presented by the child were violent vomiting and purging coming on after breakfast, followed by death in the afternoon of the same day. The suspected person was proved to have purchased both sulphate of copper and sulphate of iron not long before, and was seen by the other children to mix a blue liquid for drink after the symptoms began. Copper was naturally suspected, but there was not any found, either by the Messrs. Dewar, who conducted the first analysis, nor afterwards by Dr. Christison; but there was obtained a large quantity of iron, partly in the soluble condition, but chiefly in the form of a compound insoluble in water. This appeared to be in a great measure sulphuret of iron, formed by the decomposition of the sulphate, through means of the sulphuretted hydrogen and ammonia, disengaged during the decay of the body, for water which had acted on the contents and textures of the alimentary canal, presented evidence of a much larger quantity of sulphuric acid than would have arisen from the ordinary contents: and the whole course of the mucous coat from the mouth to the anus, was thickly lined with a layer of jet-black mucus. The tissues of the stomach presented everywhere the same color. Iron was also found largely in numerous brown stains on the child's clothes, and the apron worn by the person suspected to have administered the poison. Messrs. Dewar made some experiments with sulphate of iron, from which they came to the conclusion arrived at by Orfila and Smith, that two drachms retained in the stomach by a ligature on the œsophagus will cause death in a few hours. The process employed for detecting the iron, consisted in incinerating the contents and textures, acting on the residue with diluted nitric acid aided by heat, adding an excess of ammonia, and transmitting sulphuretted hydrogen. The ammonia separates a yellowish precipitate of sesqui-oxide of iron, and does not render the liquid blue, if there be not any copper. The sulphuretted hydrogen then produces black sulphuret of iron.—*Ibid.*

